# Postbiotic *Parabacteroides Distasonis* Supplementation Enhances Intestinal and Skeletal Muscle Function in Aged Mice

**DOI:** 10.14336/AD.2025.0188

**Published:** 2026-04-22

**Authors:** Pablo Morgado-Cáceres, Hernán Huerta, Cristian Bergman, Reinaldo Figueroa, Paula Farias, Gabriel Quiroz, Ute Woehlbier, Karen Mella, Osmán Díaz-Rivera, Sergio Linsambarth, Paulina Calderón-Romero, Felipe A. Court, Denisse Sepulveda, Daniela Sauma, Patricia Luz-Crawford, Anibal A. Vargas, Catalina Gonzalez-Seguel, J. César Cárdenas, Alenka Lovy

**Affiliations:** ^1^Center for Integrative Biology, Faculty of Sciences, Universidad Mayor, Santiago, Chile.; ^2^Geroscience Center for Brain Health and Metabolism, Santiago, Chile.; ^3^Buck Institute for Research on Aging, Novato, CA 94945, USA.; ^4^BNI, Biomedical Neuroscience Institute, Santiago, Chile.; ^5^Department of Biology, Faculty of Sciences, Universidad de Chile, Santiago, Chile.; ^6^Centro de Investigación e Innovación Biomédica, Universidad de los Andes, Santiago, Chile.; ^7^IMPACT, Center of Interventional Medicine for Precision and Advanced Cellular Therapy, Santiago, Chile.; ^8^Department of Chemistry and Biochemistry, University of California, Santa Barbara, California 93106, USA.; ^9^Center for Vision Research and the Department of Ophthalmology and Visual Sciences, SUNY Upstate Medical University Syracuse, NY 13210, USA.

**Keywords:** Mitochondria, aging, IL-10, commensal bacteria, postbiotics

## Abstract

*Parabacteroides distasonis* (Pd), a core member of the human gut microbiota, is enriched in centenarians, suggesting a potential role in promoting organismal resilience. While Pd supplementation has been shown to alleviate cancer and inflammatory diseases, its ability to mitigate the decline associated with aging remains unexplored. Here, we demonstrate that postbiotic Pd supplementation induces multiple beneficial effects in 18- and 26-month-old mice following three months of treatment. Pd-treated mice exhibit lower blood glucose levels and increased ketone body production. In the gut, Pd reduces colon shortening observed in aged control mice and decreases the inflammatory mediator NFκB, in the colonic mucosa. Microbiome analysis further reveals enhanced gut microbiota diversity in Pd-supplemented mice. Additionally, FITC-dextran permeability assays indicate improved intestinal barrier function. Cell culture experiments in HCT116 colon cell line show that Pd reduces oxygen consumption and promotes mitochondrial networking, accompanied by upregulation of PGC1α and CHOP, suggesting a mitohormetic response. Beyond metabolic and gut-related benefits, Pd supplementation enhances skeletal muscle strength in both 18- and 26-month-old mice. Proteomic analysis of gastrocnemius muscle reveals that Pd increases the expression of mitochondrial proteins associated with mitochondrial fitness and survival. Notably, Pd-supplemented mice challenged with a high-fat diet gain weight at a slower rate, while maintaining better skeletal muscle coordination and strength. In summary, our findings suggest that postbiotic Pd supplementation enhances metabolic health, reduces inflammation, improves mitochondrial function, and preserves muscle strength in aged mice. These results position Pd as a promising therapeutic tool for promoting healthy aging and combating aging-related diseases.

## INTRODUCTION

The intricate interplay between gut microbiota and host health has garnered significant attention for its potential role in modulating the aging process and associated health outcomes. Notably, the diversity of gut microbiome has been linked to various aspects of aging, with reduced microbial diversity associated with frailty. Notably, centenarians exhibit a highly diverse microbiome, correlating with successful aging and reduced chronic disease incidence [1, 2]. Interestingly, numerous studies have shown that probiotic strains, when supplemented in the diet, have improved the physiology of organisms, specifically enhancing muscle strength [3–6]. This observation underscores the potential of certain microbial genera, in promoting healthy aging through the so-called gut-muscle axis, a complex network of hormonal, metabolic, and unknown mediators connecting gut microbiota and muscle function [7]. Fielding et al., 2019 demonstrated this connection by transferring fecal samples from older adults with varying muscle functions into germ-free mice, revealing significant differences in muscle strength [8]. Furthermore, specific commensal bacterial species, such as *Faecalibacterium*, have been linked to enhanced muscle strength [9, 10] while *Bacteroides* species correlate with improved cardio-respiratory fitness and walking speed, which are key indicators of healthy aging [11–13]. In fact, *Bacteroides* and *Parabacteroides* are increased in centenarians, suggesting an important role of these genera in human resilience [14]. In particular, the genus *Parabacteroides* is composed of only four species in humans [15], *Parabacteroides distasonis* (Pd) being one of the 18 core members in the gut microbiota [16]. Live Pd supplementation has been shown to block colon tumor formation in high-fat diet-fed azoxymethane-treated mice [17], to alleviate rheumatoid arthritis [18] and to ameliorate hepatic fibrosis [19] through a mechanism that involves Pd metabolites with anti-inflammatory activity. Additionally, Pd membranous fraction reduces the severity of intestinal inflammation in murine models of acute and chronic colitis induced by dextran sulphate sodium (DSS) presumably by reducing several proinflammatory cytokines and stabilizing the intestinal microbial ecology [20]. However, whether Pd supplementation improves aging is still unclear. Moreover, whether the preparation of non-living Pd bacteria can still benefit the host during aging remains untested. Inanimate/dead microorganisms and/or their components that bring health benefits to the host have recently been defined as postbiotics [21]. Postbiotic preparations have been shown to improve the immune responses, skeletal muscle function and gut health among other effects [22–24]. In addition, postbiotics have the advantage of circumventing the technical challenge of working with live bacteria (probiotics) such as colonization efficiency and keeping the microorganisms viable and stable [25].

Emerging evidence suggests that mitochondria may serve as crucial mediators between the microbiome and host communication [26–31]. Mitochondria, originally autonomous bacteria approximately 1.5 billion years ago [32], play a pivotal role in cellular fate by regulating cellular bioenergetics, calcium (Ca^2+^) homeostasis, cell cycle, immune responses, pathogen recognition, apoptosis, autophagy and stemness [33], in addition to being the cornerstone of cellular metabolism [34]. Alterations in mitochondrial function in response to microbial cues have been implicated in enhancing the host's resistance to oxidative stress, influencing longevity, modulating inflammatory states, and impacting skeletal muscle function [35, 36]. Some of these alterations such as the increase in reactive oxygen species (ROS) and decreased respiration can instigate mitohormesis, a phenomenon that enhances cellular resilience through adaptive stress responses [37].

Here, we aimed to explore whether a postbiotic Pd supplementation improves aging through a mechanism that implies changes in mitochondrial function and a hormetic response in the colon and the skeletal muscle system.

## MATERIALS AND METHODS

### *Parabacteroides distasonis* (Pd) preparation

*Parabacteroides distasonis* (Pd, ATCC#8503) was generously provided by Dr. Jimmy Crott (Boston University). For details on Pd culturing and preparation, please refer to Koh et al., [38]. Notably, culturing the Pd preparation did not yield any live colonies, confirming the absence of viable bacteria in this formulation.

### Mouse Supplementation *with Parabacteroides distasonis* and colon harvest

Animal care and procedures were approved by the Animal Ethics Committee of the Universidad Mayor. Animal welfare was monitored by a veterinarian, who conducted daily assessments of spontaneous behavior and physical appearance, as well as weekly evaluations of body weight and behavioral responses to handling. 5-, 18- and 26-month-old C57BL/6J mice (female and male) were obtained from Jackson Laboratory and maintained at the Universidad Mayor animal facility. Mice were divided into two groups, each with an equal number of males and females, and housed under a 12:12 light/dark cycle at a controlled temperature of 21–24 °C. Both groups had *ad libitum* access to food. The control group received a standard powdered diet (Prolab RMH 3000), composed of 59% kcal from carbohydrates, 14% kcal from fat, and 26% kcal from protein. In the experimental group, the same standard diet was supplemented with 0.04% w/w Pd powder. Food was changed every two days to ensure freshness. Based on previous measurements in our lab, a single mouse consumes approximately 5 g of food per day, corresponding to an estimated daily intake of 2 mg of Pd. After the 3-month feeding period, all animals were euthanized for tissue collection. Whole colons were washed in phosphate-buffered saline and their length was determined from the cecum to the rectum. Then, the colons were cut longitudinally, and the mucosa scraped and kept frozen at -80°C for Western blot analysis. The cecal content was collected and stored at -80°C for analysis of microbial population.

### Intestinal permeability assay

For this assay, a cohort of five mice was gavaged with 10 mg of powdered Pd dissolved in 200 µL of PBS once a week for three months. The control group received 200 µL of PBS alone. At the end of the 3-month Pd supplementation period, the mice were fasted for 4 hours, after which they were gavaged with a single dose of 4-kDa FITC-dextran (Sigma-Aldrich) at 0.6 mg/g body weight to determine intestinal permeability as described [39]. Briefly, following a resting period of 4 hours after the gavage, blood samples were collected from the tail vein of each mouse. The blood samples were transferred into non-heparinized tubes and allowed to clot at room temperature for 2 hours. Serum was separated by centrifugation at 10,000 × g for 10 minutes and subsequently diluted 1:5 (v/v) in PBS. Fluorescence was measured using an FLUOstar® Omega microplate reader (BMG Labtech) with an excitation wavelength of 485 nm and an emission wavelength of 528 nm.

### Blood Measurements

Blood collection was performed between 7:00–8:00 a.m. by a small cut on the tip in non-fasting conditions. Glycemia, β-HB and lactate levels were determined at the end of the 3 months period using a Freestyle NEO monitor system (Abbott, IL, USA). All measurements were performed following the manufacturer’s instructions.

### High fat diet

Dietary treatment: sixteen C57BL/6J mice (female) were divided into two groups (eight mice each), that were fed a high fat diet (HFD, 60% kcal lipids, 19.6% kcal protein, and 16.6% kcal carbohydrates) *ad libitum* for a month. After that, one group received a posbiotic Pd supplement combined with the HFD for three more months. To ensure the consumption of the postbiotic Pd supplement we used a long-term voluntary intake protocol to decrease variability in consumption that is based in the use of hazelnut spread [40]. Briefly, Individual mice were carefully removed from their original shared cage and placed in an individual cage where they were trained (2 to 4 days) to eat commercial hazelnut spread out on the wall of the cage. After being trained, 400mg/kg of postbiotic Pd supplement was mixed with 1g of hazelnut spread (once a week, for 4 months). Control mice were exposed only to the hazelnut spread.

### Rotarod

The test was performed to evaluate motor skills using Rotarod LE8200 Panlab Harvard Apparatus (Barcelona, Spain) in two consecutive days using a described protocol [41]. On the first day, during the training session, all the animals were placed on rotating lines with a constant speed of 4 rpm by 1 min, 3 times. Later, on the second day (test session), all mice were placed on rotating lines, 3 times, with acceleration set up of 4-40 rpm for 5 min, measuring the time to fall in sec. This evaluation was performed after the end of three-month Pd administration.

### Grip test

The 4-leg grip test evaluated strength using an M&A Instruments B00EE2FCDA dynamometer vertically by pulling the mouse down by the tail [42]. The training session consisted of the mouse successfully maintaining itself with all 4 legs by pulling its tail constantly for 10 seconds. On the test day, the maximum force was evaluated in 3 repetitions in N (Newton, k*m/s^2^). This evaluation was performed after the end of three-month Pd administration.

### Microbiota Analysis by 16S rRNA Genes Sequencing

DNA extraction from cecal content was performed using the Quick-DNA Fecal/Soil Microbe Miniprep Kit (D6010, Zymo Research), according to the manufacturer's instructions. The DNA samples were sent to the Alkek Center for Metagenomics and Microbiome Research (CMMR), Houston, Texas, USA.), and the 16SV4 region was sequenced using Illumina sequencing (515F: GTG CCAGCMGCCGCGGTAA and 806R: GGACTACHV GGGTWTCTAAT) [43]. The DADA2 and Phyloseq packages were used for analysis in R 4.1.2 in RStudio. Standard parameters were used for data processing. ASV taxonomy was assigned using the SILVA V138 database as a reference. Unclassified archaea and bacteria were excluded from the analysis. Centered Log Ratio (CLR) transformation was included for ASV matrix analyses for both the Shannon index and Principal Component Analysis (PCA). Statistical analysis was performed with R, using Wilcoxon test.

### Serum cytokines

Mouse blood was collected by cardiac puncture immediately after euthanasia. The blood was received in non-heparinized tubes and allowed to clot for 2 hours at RT. After this period, the blood was centrifuged at 10,000 × g for 10 min and the serum was collected. The inflammatory cytokine levels in the mouse serum samples were quantified using the BD™ Cytometric Bead Array (CBA) Mouse Inflammation Kit (BD Biosciences, Cat# 552364) according to the manufacturer’s instructions. The data were acquired on a BD FACSCanto™ II (BD Biosciences) flow cytometer.

### Cytokine Quantification by ELISA

IL-10 quantification was measured in culture supernatants from HCT116 cells pretreated for 24h with Pd by enzyme-linked immunosorbent assay (ELISA) following manufacturing instructions (R&D Systems).

### Protein extraction for nLC-MS/MS

Proteins were extracted using 100 μL of lysis buffer (50 mM HEPES, pH 8, 1% (wt/vol) Triton X-100, 1% (vol/vol) NP-40, 1% (vol/vol) Tween 20, 1% (wt/vol) deoxycholate, 5 mM EDTA, 50 mM NaCl, 1% (vol/vol) glycerol, 1X complete protease inhibitor, and 5 mM DTT), then they were incubated for half an hour at 60° C, then the sample was homogenized by ultrasound for 2 min with cycles of 10 seconds at 40% intensity, subsequently it was alkylated using 20 mM of iodoacetamide in 25 mM of ammonium bicarbonate and incubate in darkness for 30 min. The protein extract was subjected to cleaning using the chloroform/methanol method. 1 vol of the protein extract was incorporated into 5 volumes of 100% methanol and a volume of 100% chloroform (vol/vol) was added then 3 volumes. of milliQ water, centrifuged at 15,000 x *g* for 5 min, observing a protein disc, the soluble phase is eliminated and then the disc is washed with 400 µL of 100% methanol 4 times, were dried in a rotary concentrator at 2,000 rpm overnight at 40°C.

The samples were resuspended in a maximum of 100 µL of 8M Urea and 25 mM ammonium bicarbonate. The mixture was incubated for 5 minutes at 25°C and vortexed several times. The samples were sonicated on ice with 10-second on/off pulses at 90% amplitude and incubated on ice for 5 minutes. Centrifugation was performed at 19,000 x g for 15 minutes at 4°C, and the supernatant was transferred to a new tube. The proteins were immediately quantified using the Qubit Protein Assay reagent (#Q33212, Invitrogen). Digestion was carried out with sequencing-grade trypsin (#V5071, Promega) at a 1:50 protease/protein (w/w) ratio and incubated for 16 hours at 37°C. Digestion was stopped by adding 10% formic acid. The samples were then cleaned using EVOTips following the supplier's instructions.

### nLC-MS/MS timsTOF PRO 2 analysis

The cleaned peptides were injected into a EVOSEP ONE nano liquid chromatography system (EVOSEP System), Peptides (200 ng of digest) were separated within 30SPD gradient on a reversed-phase column Bruker pepsep 15 (15 cm x 150 µm i.d. C18 1.6 µm) (Bruker, Daltonics) with 50°C. Mobile phases A and B were water and acetonitrile with 0.1 vol% FA, respectively.

All samples were analyzed on a hybrid trapped ion mobility spectrometry (TIMS) quadrupole time-of-flight mass spectrometer (TIMS-TOF Pro, Bruker Daltonics) via a CaptiveSpray nano-electrospray ion source [44]. The MS was operated in data-dependent mode for the ion mobility-enhanced spectral library generation. The accumulation and ramp time was 100ms each and recorded mass spectra in the range from m/z 100–1700 in positive electrospray mode. The ion mobility was scanned from 0.8 to 1.3 Vs/cm^2^. The overall acquisition cycle of 0.5 s comprised one full TIMS-MS scan and 4 parallel accumulation-serial fragmentation (PASEF) MS/MS scans.

### Database Searching

We used FragPipe computational platform (version 15) with MSFragger (version 3.2), [45, 46], Philosopher (version 3.4.13), [47] and EasyPQP (https://github.com/grosenberger/easypqp; version 0.1.9) components to build spectral libraries. Peptide identification from tandem mass spectra (MS/MS) was done using MSFragger search engine, using either raw (.D) files as input. Protein sequence databases *Mus musculus* (UP000000589) from UniProt (reviewed sequences only; downloaded on Feb. 15, 2021) and common contaminant proteins, containing in total 34650 (*Mus musculus*) sequences were used. Reversed protein sequences were appended to the original databases as decoys. For the MSFragger analysis, both precursor and (initial) fragment mass tolerances were set to 20 ppm. Enzyme specificity was set to ‘stricttrypsin’, and fully enzymatic peptides were allowed. Up to two missed trypsin cleavages were allowed. Oxidation of methionine, acetylation of protein N-termini, -18.0106 Da on N-terminal Glutamic acid, and -17.0265 Da on N-terminal Glutamine and Cysteine were set as variable modifications. Carbamidomethylation of Cysteine was set as a fixed modification. Maximum number of variable modifications per peptide was set to 3. The final spectral library was filtered to 1% protein and 1% peptide-level FDR.

### Bioinformatics Analysis

The output quantification reports from MSFragger were exported and processed in the R statistical environment. Intensity values for each run were normalized by adjusting the medians. Missing values were imputed for each condition using the missForest algorithm, applying the criterion that the intensity value should be present in at least 60% of the samples per analyzed category. Significant differential protein expressions were determined using a Bayesian t-test. Any protein associated with a p-value < 0.05 was considered significant.

Then, the proteomic dataset including UniProt identifiers, logFC and p values of identified proteins in Mass spectrometry was submitted to Ingenuity Pathway Analysis (IPA, QIAGEN Inc., https://digitalinsights.qiagen.com/IPA). Only DEPs that passed a p value filter of 0.05 were considered for analysis. A core analysis was performed with the following settings: (i) indirect and direct relationships between molecules, (ii) based on experimentally observed data, and (iii) all data sources were admitted from the Ingenuity Knowledge Base [48]. Mouse MitoCarta 3.0 database was used to determine mitochondrial proteins [49].

### Cell culture and Pd treatment

Human colorectal cell line HCT116 (ATCC) was maintained at 37ºC (95%/5% air/CO_2_) in DMEM media (GIBCO) supplemented with 10% (v/v) FBS. Skeletal muscle cell line Hskm was maintained at 37ºC (95%/5% air/CO_2_) in αMEM/F12 supplemented with 200mM L-glutamine, 1% FBS, 1mg/ml EGF, 0.1 mg/mL βFGF, 100UI/mL insulin and 10mM dexamethasone. All cells were grown in the presence of 100 U mL-1 penicillin, 100 µg mL-1 streptomycin and 0.25 µg mL-1 fungizone (Gibco) at 37ºC (95%/5% air/CO_2_). Based on previously published data demonstrating that incubation with 25 µg/mL of postbiotic Pd for 24 hours alters intracellular protein expression [38], we sought to determine the minimal effective concentration in our system. Our findings indicate that 12.5 µg/mL of postbiotic Pd for 24 hours is the lowest concentration capable of inducing measurable effects.

### Confocal live imaging and mitochondrial morphology

Live cell imaging was performed on a Nikon A1R confocal microscope equipped with Perfect Focus using a 63X plan apo lens/NA 1.4. To determine mitochondrial morphology the cell lines were loaded with tetramethylrhodamine methyl ester (TMRE, 8 nM, Life Technologies) for 30 min at 37°C and 5% CO_2_. Analysis of fluorescence intensity was performed using Fiji. Mitochondrial network analysis was performed with Fiji plugin called MiNa, developed in the Stuart Lab [50].

### Extracellular flux analysis

Oxygen consumption rate (OCR) was assessed in an extracellular flux analyzer XF^e^96 (Agilent Technologies™, CA, USA) as described previously [51]. Briefly, either HCT116 or Hskm were seeded on XFe96-well plates (15,000 cells and 20,000 cell per well respectively) and incubated for 24h with the corresponding treatment (Pd or IL-10) at 37°C in 5% CO_2_ atmosphere. The following day, the culture medium was replaced with assay media (unbuffered DMEM supplemented with glutaMAX®, 10 mM glucose and 1 mM pyruvate, pH 7.4) 1 hour prior to the assay and left for the duration of the experiment. After establishing the baseline OCR, cells were sequentially challenged with oligomycin (1 μM), FCCP (250 nM for HCT116 and 500 nM for Hskm) and rotenone + Antimycin A (both 1 μM) to reveal basal and maximal respiration. The data were normalized by protein content.

### Western blotting

Colon mucosa samples or cells in culture were lysed with Cytobuster protein extraction reagent (Novagen) supplemented with protease (Sigma) and phosphatase inhibitors (complete PhosSTOP, Roche). Protein extracts were separated in 10% or 15% SDS-polyacrylamide gels and transferred to PDVF membranes (Millipore). Blocking was performed at room temperature for 1 h in 5% fat-free milk, and membranes were incubated overnight at 4°C with primary antibody: NF-κB p65 (C22B4) #4764 (Cell Signaling), PGC1α #ab191838 (Abcam), CHOP (L63F7) #2895 (Cell Signaling), VDAC1 #ab3472 (Abcam). Then for 1 h at room temperature with a secondary antibody conjugated to horseradish peroxidase; Anti-rabbit secondary antibody HRP (#31460), anti-mouse secondary antibody HRP (#31430) both from Thermo Fisher Scientific. Chemiluminescence detection used ECL-plus reagent (Pierce) and a series of time exposure images were acquired with a FluorChem Q system (ProteinSimple). To determine protein loading across gels, membranes were striped and re-blotted with either β-actin or GADPH. Fiji was used for densitometric analysis.

### Statistical analysis

All statistical analyses were performed using GraphPad Prism (GraphPad Software Inc.) and R (R Foundation for Statistical Computing). The normal distribution of the data was determined by Shapiro-Wilk test. Comparisons between two groups were conducted using either the unpaired Student's t-test (for normally distributed data) or the unpaired Mann-Whitney test (for non-normally distributed data). Multiple group comparisons were performed using one-way ANOVA followed by Tukey’s post hoc test. Longitudinal body weight measurements across different groups were analyzed using two-way ANOVA followed by Bonferroni’s post hoc test. All graphs represent mean ± standard error of the mean (SEM). Statistical significance was set at p < 0.05, with significance levels indicated as follows: *p ≤ 0.05, **p ≤ 0.01, ***p ≤ 0.001, and **p ≤ 0.0001.

**Figure 1.**
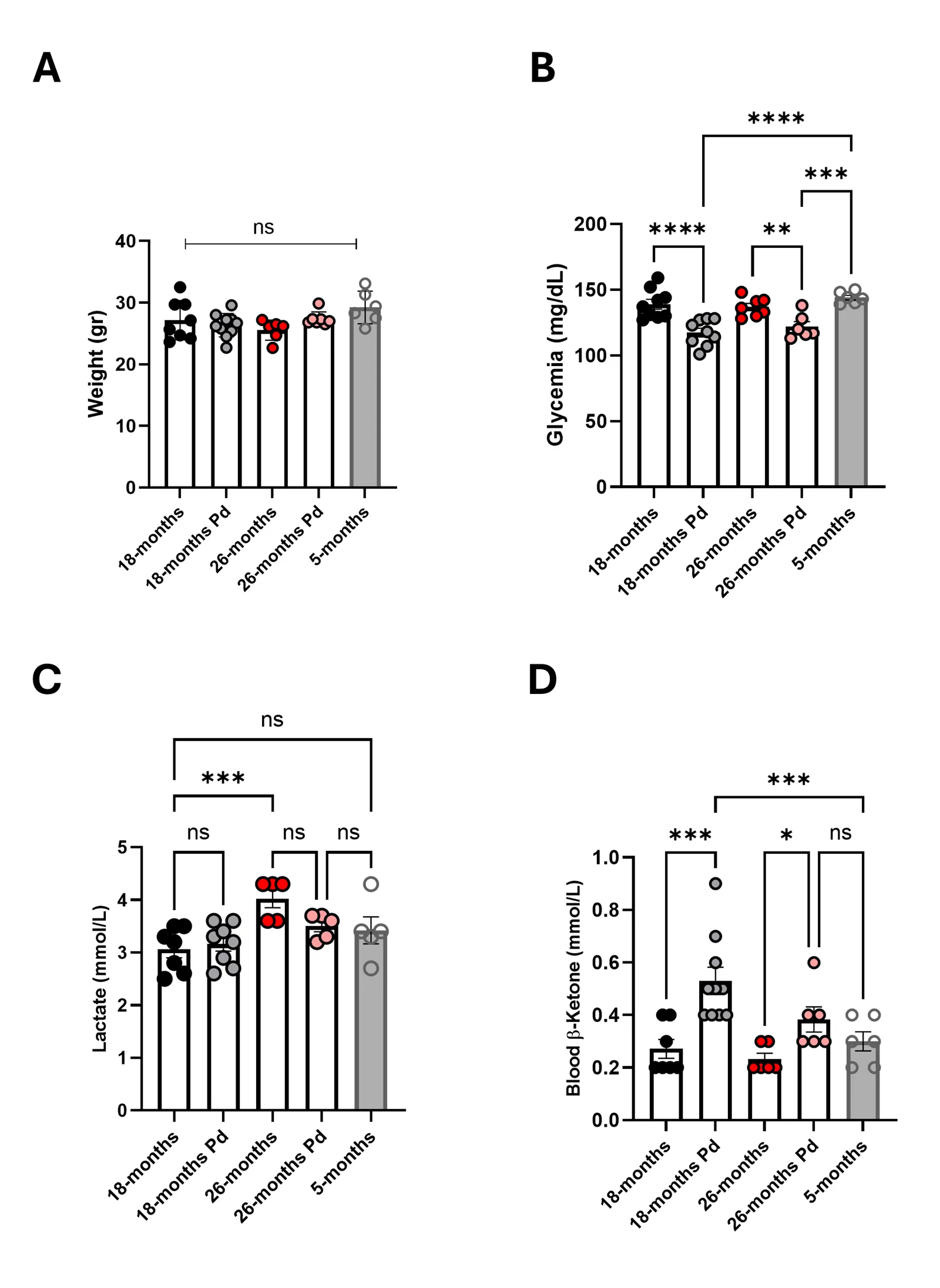
**Effects of postbiotic Pd supplement on weight, glycemia, lactate and ketone body levels.** Total body weight in 18- and 26-month-old mice after three months of postbiotic Pd supplementation and control untreated 5-month-old mice at the end of the experiment **(A)** Blood measure of glucose **(B)**, lactate **(C)** and ketone bodies **(D)** in 18- and 26-month-old mice after three months of postbiotic Pd supplementation and control untreated 5-month-old mice. N = 8-9 for the 18-month-old group, 5-6 for the 26-month-old group and 6 for the 5-month-old group. Data are expressed as MEAN ± SEM. ****p≤0.0001, ***p≤0.0005, **p≤0.005, *p≤0.01, ns=not significant. *One-way ANOVA with post hoc Tukey’s test*.

## RESULTS

### Supplementation with postbiotic Pd induces positive systemic metabolic changes, reduces inflammation and improves gut health in old mice.

**Figure 2.**
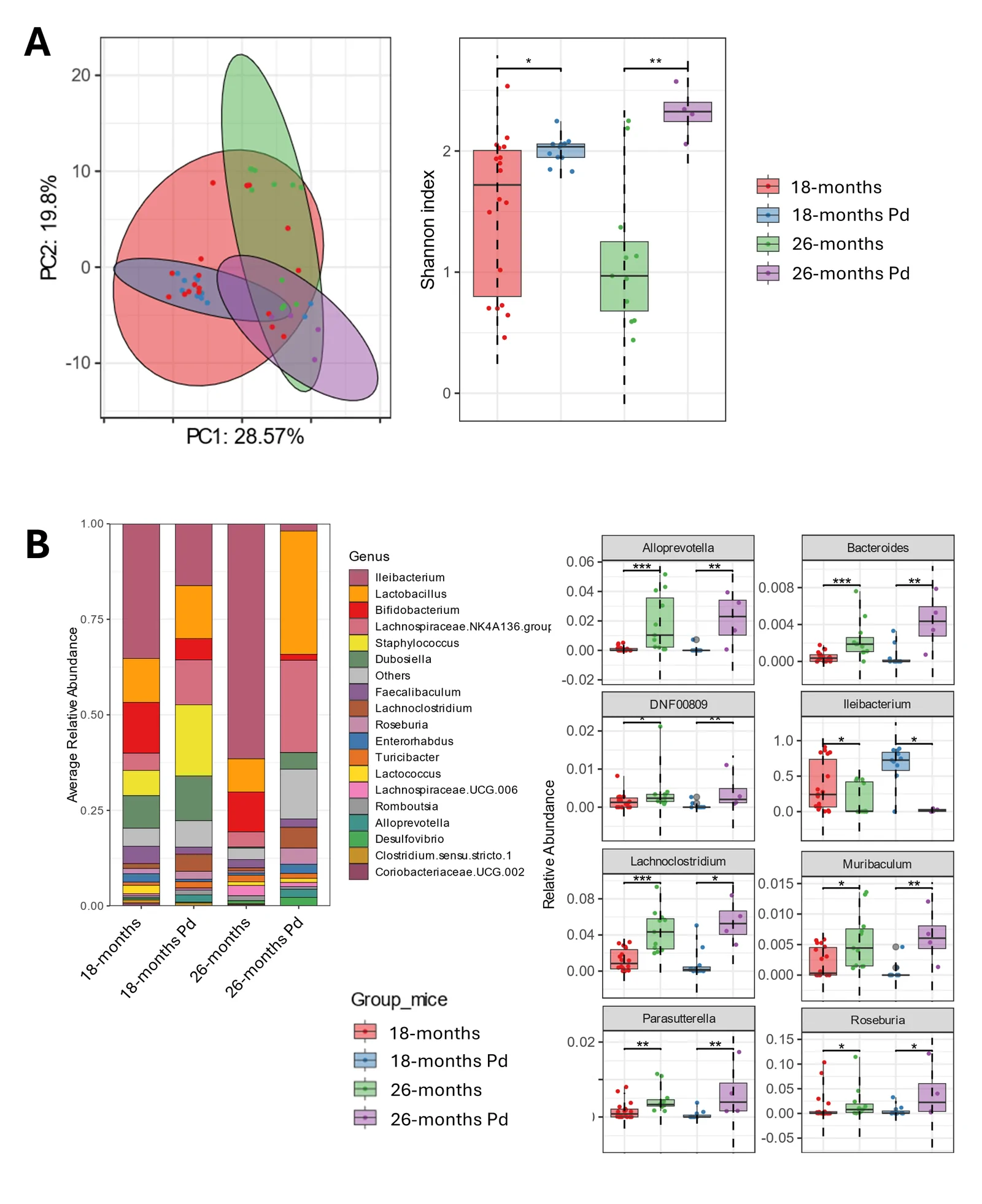
**Gut microbiota diversity and taxonomic classification of cecal content in 18- and 26-month-old mice treated with Pd.** In **(A)**, the left panel shows the beta diversity through Principal Component Analysis (PCA) using Euclidean distance on Centered log ratio (CLR) transformed data; the percentages of variance explained by the first two components are 28.57% (PCA1) and 19.8% (PCA2). The right panel shows the alpha diversity based on the Shannon index, and statistical significance among groups was evaluated using the *non-paired Wilcoxon test* (*, p < 0.05; **, p < 0.01). A total of 44 samples were analyzed, distributed as follows: CTL 18-month-old mice (n = 18; red), CTL 26-month mice (n = 11; blue), Pd 18-month-old mice (n = 11; green), and Pd 26-month-old mice (n = 4; purple). In **(B)**, the left panel presents the average relative abundance at the genus level, with the remaining genera grouped as "Others" in gray. The right panel illustrates the genera that change in relative abundance due to Pd treatment in 18- and 26-month-old mice. Red and blue correspond to 18- and 26-month control mice respectively. Green and purple correspond to 18- and 26-month-old mice treated with Pd. Statistical analyses were performed in R using the *non-paired Wilcoxon test* (*, p < 0.05; **, p < 0.01; ***, p < 0.001).

Various strains of *Parabacteroides distasonis* (Pd) have demonstrated positive effects while being supplemented in the mouse diet. Whether a postbiotic Pd preparation has any positive effects on aged mice is unknown. Here, we supplemented 18-month-old and 26-month-old C57BL/6J mice with a postbiotic formulation of Pd, consisting of lyophilized dead Pd for 3 months. At the end of this period, we determined the weight of the mice and measured non-fasting plasma glucose, ketone bodies and lactate levels. At the end of the experiment, no difference in weight was found between Pd treated and control mice (Fig. 1A). Interestingly, glucose levels in both 18- and 26-month-old Pd treated mice are lower than the control mice and even lower than the levels observed in young (5-month-old) mice (Fig. 1B). Accordingly, our results show that 26-month-old mice have higher levels of plasma lactate than 18-month-old mice. Interestingly, the Pd treatment reduces lactate levels in 26-month-old mice to the levels observed in 5-month-old mice (Fig. 1C). Moreover, we found that Pd slightly increases ketone levels at both ages (Fig. 1D). These metabolic changes may be the result of enhanced microbial diversity observed in both 18-month-old and 26-month-old mice fed with Pd (Fig. 2A).

**Figure 3.**
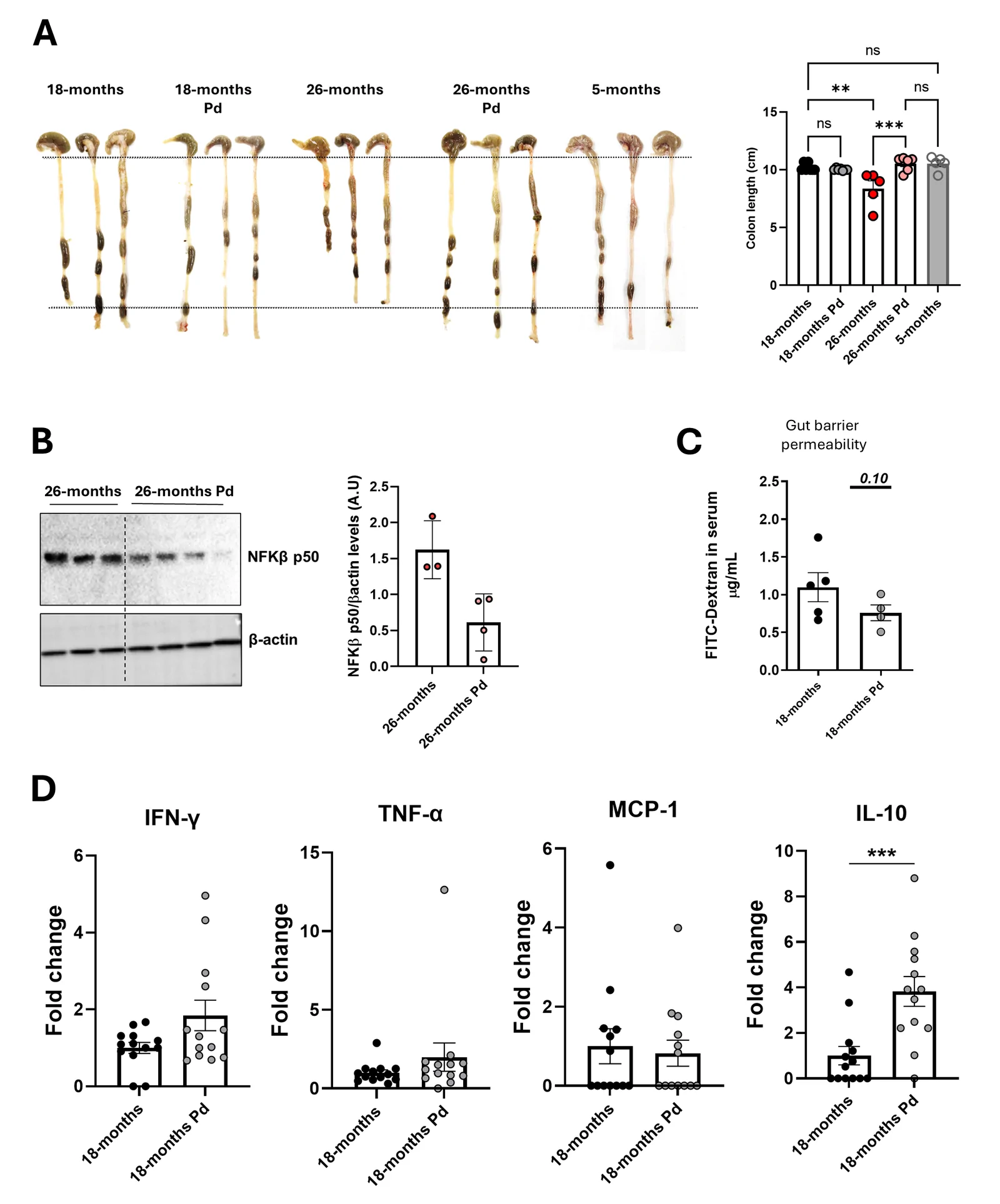
**Postbiotic Pd supplement reduces colon and systemic inflammation. (A)** Left panel- representative images of untreated control 5-month-old mice colon and 18- and 26-month-old mice colon either after three months of postbiotic Pd supplementation or control. Right panel- analysis of colon length. N = 8-9 for the 18-month-old group, 5-6 for the 26-month-old group and 6 for the 5-month-old group. Data are expressed as MEAN ± SEM. ***p≤0.0005, **p≤0.005, ns=not significant. *One-way ANOVA with post hoc Tukey’s test.* **(B)** Left panel- representative Western blot of NFκβ and β-actin as loading control in 26-month-old mice colon mucosa. Right panel- bar graph: NFκβ/β-actin levels compared to the control (arbitrary units. N=3 for the control and 4 for the Pd group. Data are expressed as MEAN ± SEM. *p≤0.05. *Mann-Whitney test*. **(C)** FITC-dextran (0.6 mg/g body weight) fluorescence determined for 4h after gavage in the serum of 18-month-old mice either on a normal diet or supplement with postbiotic Pd for three months. N=5 for the control group and 4 for the Pd group. Data are expressed as MEAN ± SEM. *Mann-Whitney test*. **(D)** Pro-inflammatory TNF-α, IFN-γ and MCP-1 and anti- inflammatory IL-10 cytokines were determined in the serum of 18-month old mice treated or not with postbiotic Pd supplement using a cytometric bead array. N = 12. Data are expressed as MEAN ± SEM. ***p≤0.0005, *p≤0.05. *t-test*.

Using Principal Component Analysis (PCA), the diversity between the different groups of 18-month-old and 26-month-old control mice partially shares the position of the samples on the graph. However, when treated with Pd, there is a shift, indicating that the composition of the gut microbiome has changed with the treatment. Additionally, the Shannon index also indicates changes in the diversity and/or abundance of the intestinal microbiota bacteria, showing that treatment with Pd significantly increases diversity in both 18- and 26-month-old mice. When analyzing the gut microbiota at the genus level, we observed that some genera are altered both by age and by Pd treatment (Fig 2B). Specifically, the genera *Alloprevotella*, *Bacteroides*, DNF00809, *Lachnoclostridium*, *Muribaculum*, *Parasutterella*, and *Roseburia* were significantly increased (Fig. 2B), while the abundance of *Ileibacterium* significantly decreased in Pd-fed mice compared to the control (Fig. 2B). It is important to highlight that these variations in genus are observed in both 18- and 26-month-old mice. Additionally, in old mice, other genera increased their relative abundance when administered Pd compared to the control mice, including *Acetatifactor*, *Anaerotruncus*, *Butyricicoccus*, *Colidextribacter*, *Dorea*, *Eisenbergiella*, *Enterorhabdus*, *Helicobacter*, *Intestimonas*, *Lactobacillus*, and *Marvinbryantia*, with only *Bifido-bacterium* decreasing (*data not shown*).

**Figure 4.**
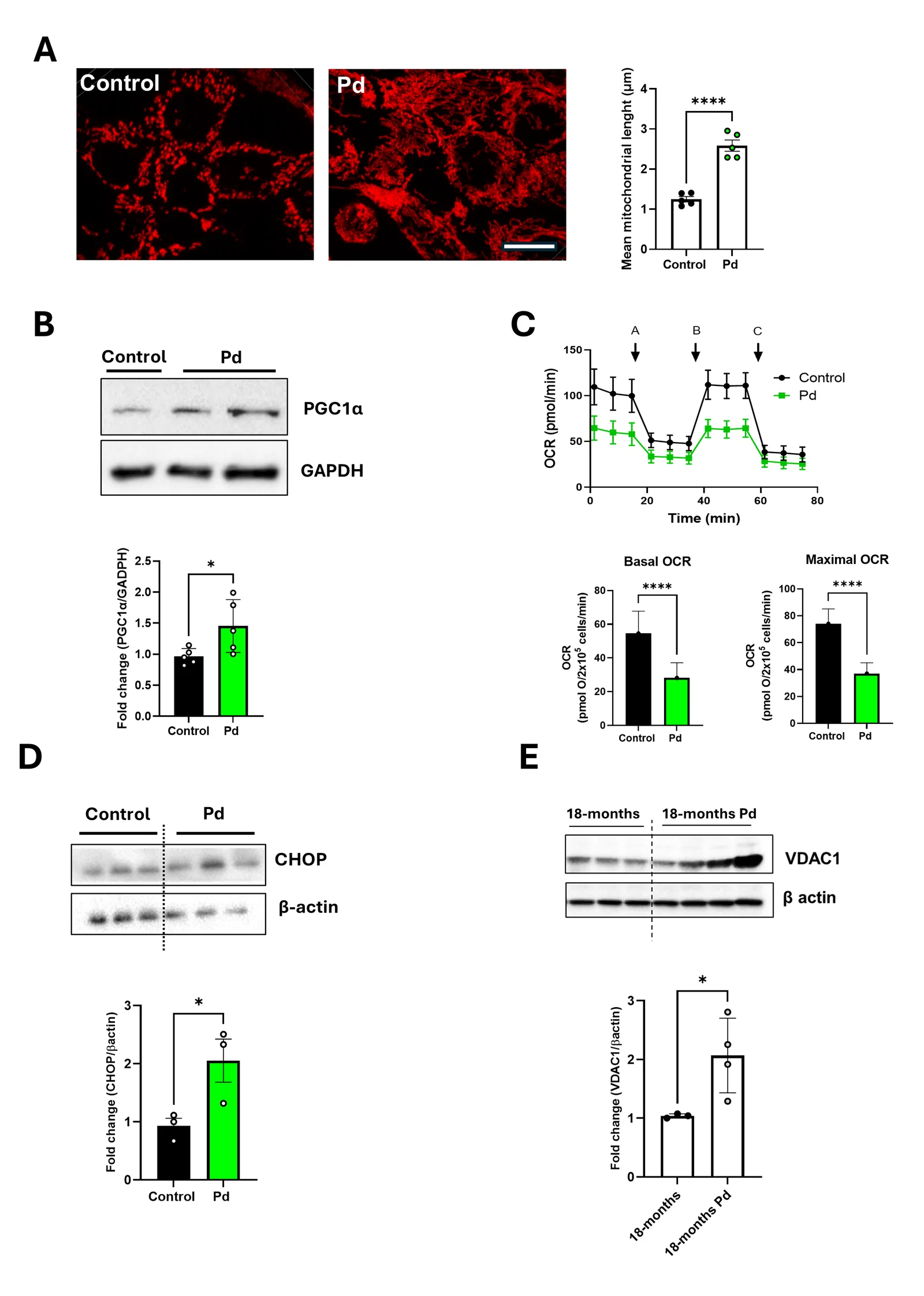
**Postbiotic Pd modifies mitochondria in a colon cell line. (A)** Right panel-representative images of HCT116 treated or not with postbiotic Pd for 24h and labeled with TMRE to determine mitochondrial morphology. Bar = 10 µm. Left panel- bar graphs of the mitochondrial length analysis. Data are expressed as MEAN ± SEM of five independent experiments ****p≤0.0001. *Mann-Whitney test*. **(B)** Upper panel- representative Western blot of PGC1α and GADPH as a loading control in HCT116 cells treated or not with postbiotic Pd. Bottom panel- Bar graphs represent quantification of PGC1α/GADPH expressed as MEAN ± SEM of five independent experiments. ∗p < 0.05. *Mann-Whitney test*. **(C)** Upper panel- representative Seahorse trace of HCT116 cells treated or not with postbiotic Pd. A; oligomycin (1 µM), B;FCCP (250 µM), C; rotenone plus antimycin A (1 µM each). Bottom panel- basal and maximum OCR of HCT116 cells treated or not with postbiotic Pd. MEAN ± SEM of three independent experiments with 10 replicates each. ***P < 0.001 compared to control. *Mann-Whitney test.* **(D)** Upper panel- representative Western blot of CHOP and β-actin as a loading control in HCT116 cells treated or not with postbiotic Pd. Bottom panel- bar graphs represent quantification of CHOP/β-actin expressed as MEAN ± SEM of 3 independent experiments. ∗p < 0.05. *Mann-Whitney test*. **(E)** Upper panel- representative Western blot of VDAC1 and β-actin as a loading control in colon mucosa samples from 26-months-old mice treated or not with postbiotic Pd. Bottom panel- bar graphs represent quantification of VDAC1/β-actin expressed as MEAN ± SEM. Note that β-actin blot in Figure 3B is the same as in this figure, as the same membrane was stripped and re-probed for VDAC1. N = 3 in the control group and 4 for the Pd treated group. ∗p < 0.05. *Mann-Whitney test*.

Aging is usually accompanied by a low-grade chronic inflammation known as inflammaging [52], that is commonly associated with increased intestinal epithelial permeability (leaky gut). Thus, to further characterize the effects of Pd in aging mice, we measured the length of the colons which are known to shorten with inflammation [53–55]. In 18-month-old mice no differences in the length of the colon were observed between control and Pd treated mice. However, in 26-month-old mice, we observed a consistent shortening in the colon of control mice, which is restored by Pd to the levels observed in young (5-month-old) mice (Fig. 3A). Presumably, the effect of Pd on conserving colon length in 26-month-old mice was due to the anti-inflammatory properties of Pd described previously [38]. Indeed, Pd treated 26-month-old mice show in the colon mucosa reduce levels of the inflammatory marker nuclear factor kappa-light-chain-enhancer of activated B cells (NFκβ) (Fig. 3B). As inflammation is closely linked with the permeability of the gut barrier, we tested the integrity of the barrier by applying FITC-dextran via gavage. Unfortunately, 26-month-old mice were not able to tolerate the procedure. Nevertheless, we hypothesized that the loss of gut barrier integrity precedes inflammation and the shortening of the colon and could be observed in 18-month-old mice. Thus, mice treated with Pd exhibited a clear trend toward reduced FITC-dextran passing into the bloodstream, suggesting improved intestinal barrier integrity compared to control-aged mice (Fig. 3C). Notably, a significant reduction in FITC-dextran permeability was observed in 18-month-old mice that received a precisely measured Pd gavage for three months (Supplementary Fig. 1A). We believe that a better barrier integrity should result in a reduction of general inflammation. Thus, we determined the presence of several interleukins in the blood using a Cytometric Bead Array (CBA) mouse inflammation kit. Notably, the tumor necrosis factor-alpha (TNFα), interferon-γ (IFN-γ) and the monocyte chemoattractant protein-1 (MCP-1) levels did not change with Pd, but plasma levels of interleukin 10 (IL-10), an anti-inflammatory cytokine was increased [56] (Fig 3D). Altogether, our data suggests that a postbiotic Pd supplementation improves metabolic fitness, reduces inflammation and improves gut health in old mice.

### Pd impacts mitochondrial function in a colon cell line

To explore whether Pd affects mitochondria, we treated the intestinal epithelial cell line HCT116 with 12.5 µg/mL of postbiotic Pd for 24 h and examined mitochondrial morphology. We found a much more extensive networking of mitochondria in the cells treated with Pd (Fig. 4A). In addition, we observed an increase in the peroxisome proliferator-activated receptor gamma coactivator 1-alpha (PGC1α), the master regulator of mitochondrial biogenesis induced by Pd (Fig. 4B), which suggests an increase in the number of mitochondria and a metabolic rewiring. Next, we thought to determine whether mitochondrial function is affected by measuring oxygen consumption rates (OCR). To our surprise, basal and maximal OCR rates decreased after treatment with Pd (Fig. 4C). This reduction in respiration could trigger the increase of PGC1α as part of a stress response initiated to maintain cellular homeostasis. We therefore measured the expression of the stress response protein C/EBP homologous protein (CHOP) finding a significant increase induced by Pd (Fig. 4D). In addition, mitochondrial number also increased in the colonic mucosa of 26-month-old mice treated with Pd, as determined by the expression of the mitochondrial outer membrane protein voltage-dependent anion channel 1 (VDAC) (Fig. 4E).

Overall, our results suggest that Pd, directly or indirectly, modulates the mitochondrial stress response, which may mediate the benefits of postbiotic Pd supplementation.

**Figure 5.**
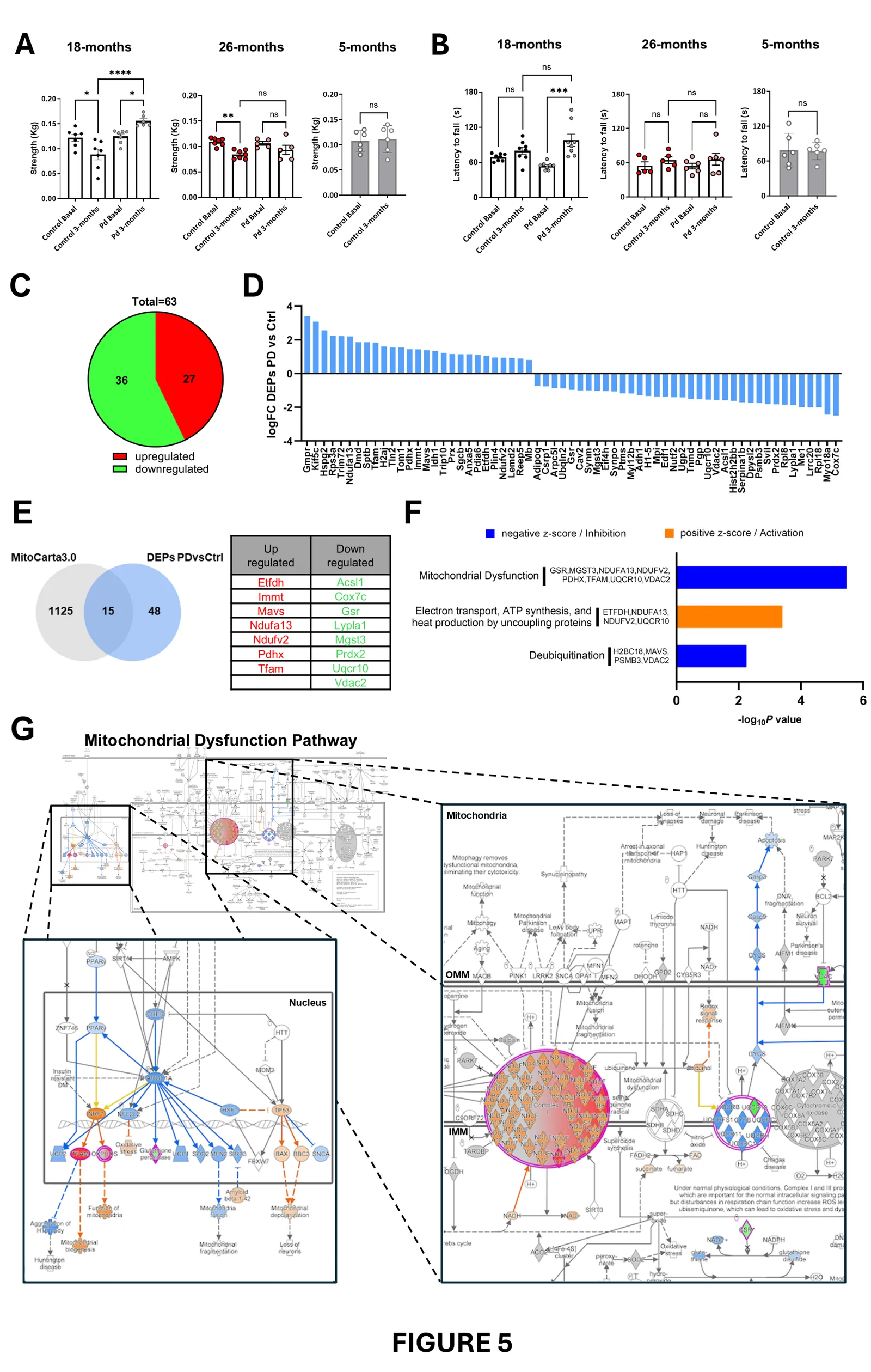
**Postbiotic Pd supplement improves skeletal muscle performance. (A)** Fore-/hindlimb (4 paws) grip strength on 18- and 26-months old mice control or treated with postbiotic Pd supplement for three months and control untreated 5-month-old mice. N = 8-9 for the 18-month-old group, 5-6 for the 26-month-old group and 6 for the 5-month-old group. Data are expressed as MEAN ± SEM. ****p≤0.0001, **p≤0.005, *p≤0.01, ns=not significant. *One-way ANOVA with post hoc Tukey’s test*. **(B)** Latency to fall on accelerating rotarod performed in 18- and 26-months old mice control or treated with postbiotic Pd supplement for three months and control untreated 5-month-old mice. N = 8-9 for the 18-month-old group, 5-6 for the 26-month-old group and 6 for the 5-month-old group. Data are expressed as MEAN ± SEM. ***p≤0.0005, *p≤0.01, ns=not significant. *One-way ANOVA with post hoc Tukey’s test.* **(C-G)** Proteomics and bioinformatic analysis suggest that mice treated with Pd display improved muscular mitochondrial function. **C**. Muscle tissue was extracted from mice treated with Pd (n=6) and control mice treated with vehicle (n=6). Mass spectrometry followed by bioinformatic analysis was performed. A total of 63 differentially expressed proteins (DEPs) were consistently detected passing the p-value 0.05 comparing groups of Pd treated versus non-treated control mice. 27 DEPs were upregulated, and 36 DEPs were downregulated. **(D)** The amplitude of expressions of all 63 DEPs is displayed. **(E)** Venn Diagram overlaying 1140 mouse proteins known to be located to mitochondria (MitoCarta3.0 database) with the 63 identified DEPs. 15 DEPs were found to locate in mitochondria, of which 7 were upregulated and 8 downregulated. **(F)** Pathway analysis using Ingenuity Pathway Analysis (IPA) suggested only 3 pathways affected by the identified DEPs. The Mitochondrial Dysfunction and Deubiquitination pathways were interpreted according to the pattern of detected DEPs as being inhibited (negative z-score, blue), while the pathway for Electron transport, ATP synthesis and heat production by uncoupling proteins was found activated (positive z-score, orange). **(G)** The most significant Pathway, Mitochondrial Dysfunction, is displayed in detail (red, detected and upregulated; green, detected and downregulated; orange, predicted activation; blue, predicted inhibition). Due to the regulatory network known to govern the pathway, predicted consequences of the detected DEPs were increased mitochondrial biogenesis and function and decreased apoptosis.

### Postbiotic Pd supplement prevents the loss of skeletal muscle strength that accompanies aging in mice

Cumulative evidence suggests that improving gut health positively impacts muscle performance [57]. Thus, we sought to determine muscle strength and motor basal coordination in 18- and 26-month-old mice treated for 3 months with Pd by the grip and rotarod assays, respectively. Pd treated 18-month-old mice significantly increased their strength after 3 months of supplementation (Fig 5A). A similar improvement in strength was observed in mice that received Pd *via* gavage for 3 months (Supplementary Fig. 1B). Moreover, this change is more significant when compared with the control group, which actually lost strength in the course of the experiments. On the other hand, the 26-month-old mice treated with Pd did not increase their strength throughout the experiment, or lost strength as the control group (Fig 5A). The basal strength levels observed in 18- and 26-month-old mice were comparable to those of 5-month-old mice. Notably, the improvement in strength observed in 18-month-old mice fed with Pd surpassed the strength levels measured in 5-month-old mice, suggesting a significant enhancement in muscle function with Pd supplementation. The rotarod experiments show that 18-month-old mice significantly improve their performance after 3 months of Pd supplementation (Fig. 5B). A similar trend was observed in mice that received Pd via gavage for 3 months (Supplemtary Fig. 1C). However, in 26-month-old mice no differences were found between the conditions (Fig 5B), which suggests that there is a window for successful intervention that impacts muscle function. Similarly, the basal performance levels observed in 18- and 26-month-old mice were comparable to those of 5-month-old mice. Notably, Pd-treated mice exhibited performance levels that exceeded those of the younger cohort, indicating a significant enhancement in functional capacity.

To gain insight into the changes occurring in skeletal muscle following Pd supplementation, we performed a proteomic analysis of gastrocnemius samples of mice treated with or without Pd. A total of 63 differentially expressed proteins (DEPs) were detected in the muscle comparing Pd-treated mice versus non-treated mice, of which 27 were upregulated and 36 were downregulated (Fig. 5 C and D). A significant number of DEPs were found to be in mitochondria (Fig. 5E). Using Ingenuity Pathway Analysis (IPA) DEPs were found to be associated mainly with 3 pathways, (i) Mitochondrial Dysfunction, (ii) Electron Transport, ATP synthesis and heat production by uncoupling proteins and (iii) Deubiquitination (Fig. 5F). The Mitochondrial Dysfunction pathway was suggested to be inhibited by IPA and therefore is displayed in detail (Fig. 5E). Interestingly, the transcription factor A (TFAM), a transcriptional regulator specifically acting on mitochondrial gene expression was significantly upregulated. On the other hand, VDAC2, which is associated with apoptosis was found downregulated [58, 59]. In summary, these results suggest that the treatment with Pd may have a positive effect on mitochondrial functioning and cell survival in the muscle.

### Postbiotic Pd supplement partially prevents the skeletal muscle decay induced by a high fat diet

**Figure 6.**
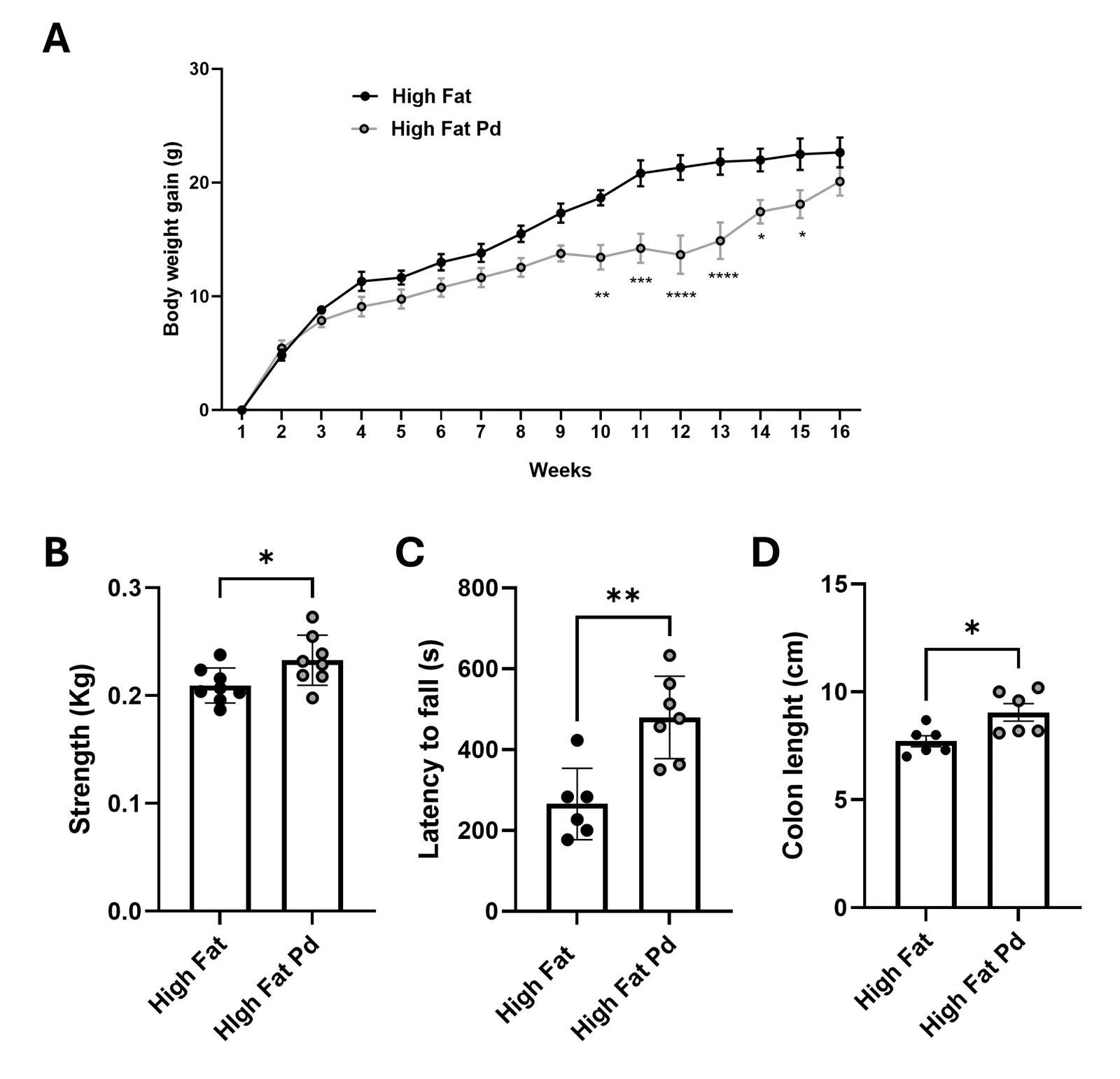
**Postbiotic Pd supplement protects skeletal muscle cell function against a high fat diet challenge. (A)** Body weight measured from the beginning of a high fat diet. 18-month-old mice were treated with a high fat diet for a month and then one group received a postbiotic Pd supplement together with the high fat diet for three months. N = 6 for the control group and 9 for the Pd treated group. ∗p < 0.05, ∗∗p < 0.005, ∗∗∗p < 0.001, and ∗∗∗∗p < 0.0001, *two-way ANOVA with post hoc Bonferroni test*. **(B)** Fore-/hindlimb grip strength on 18-months old control or treated with postbiotic Pd supplement mice. N = 8 for both groups. Data are expressed as MEAN ± SEM. *p < 0.05, *t-test*. **(C)** Latency to fall on accelerating rotarod performed in 18-months old control or treated with postbiotic Pd supplement mice. N = 7 for both groups. Data are expressed as MEAN ± SEM. **p≤0.005, *t*-*test*. **(D)** Analysis of colon length. N = 6 for both groups. Data are expressed as MEAN ± SEM. *p < 0.05, *t-test*.

Our proteomic data suggests that Pd induces changes, mostly mitochondrial, that favor homeostasis and cell survival. Thus, to prove that this was the case, we challenged a cohort of 18-month-old mice with a high fat diet in the presence of Pd. The mice received a high fat diet for a month followed by three months of a combination of high fat diet plus postbiotic Pd supplement. The control group did not receive Pd. We monitored weight gain, skeletal muscle strength and motor coordination. After 1 month of Pd supplementation, mouse weight gain was significantly slowed for about 2 months, after which the Pd treated group reached weight levels similar to those observed in the control mice (Fig. 6A). Muscle strength measurements at the end of the experiment reveal that mice which received the postbiotic Pd supplementation perform better than the controls (Fig. 6B). Similarly, balance and motor coordination were significantly better in the mice that received Pd (Fig. 6C). Overall, our findings demonstrate that Pd confers significant protection to skeletal muscle against damage induced by a high-fat diet. Whether these benefits arise directly from the observed changes in the mitochondrial proteome or result from a broader mechanism, such as reduced inflammation, remains to be elucidated. Finally, we assessed colon length in these mice and found that the shortening induced by a high-fat diet [60, 61] was prevented by Pd supplementation (Fig. 6D).

**Figure 7.**
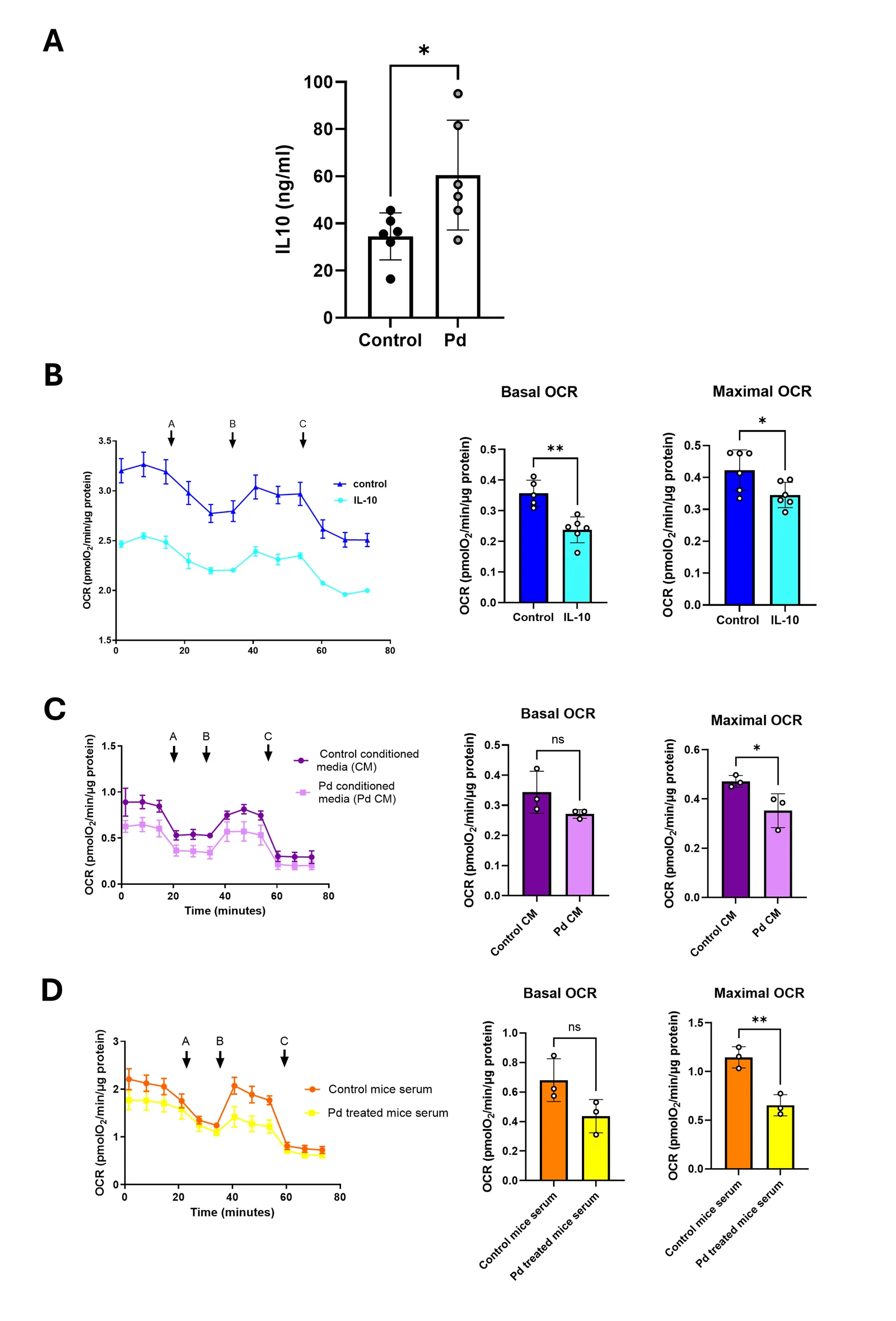
**IL-10 secreted from intestinal cells upon Pd stimulation modified mitochondrial function in skeletal muscle cells. (A)** ELISA analysis for IL-10 in conditioned media from HCT166 cell treated or not with postbiotic Pd. N = 6. Data are expressed as MEAN ± SEM. *p<0.05, *t-test*. **(B)** Left panel- representative Seahorse trace of Hskm cells treated or not with IL-10. A; oligomycin (1 µM), B;FCCP (500 µM), C; rotenone plus antimycin A (1 µM each). Right panel- basal and maximum OCR of Hskm cells treated or not with IL-10. MEAN ± SEM of four independent experiments with 3 replicates each. **p < 0.01, *p < 0.05, compared to control. *Mann-Whitney test*. **(C)** Left panel-representative Seahorse trace of Hskm cells treated with control or Pd treated cell conditioned media (CM). A; oligomycin (1 µM), B;FCCP (500 µM), C; rotenone plus antimycin A (1 µM each). Right panel- basal and maximum OCR of Hskm cells treated or not with control or Pd treated cell conditioned media (CM). MEAN ± SEM of 3 independent experiments with 3 replicates each. *p < 0.05, ns= no significant compared to control. *Mann-Whitney test.* **(D)** Left panel- representative Seahorse trace of Hskm cells treated with serum from Pd treated mice or control. Right panel- basal and maximum OCR of Hskm cells treated with serum from 3 different Pd treated mice or 3 different control mice. MEAN ± SEM of 3 independent experiments with 3 replicates each. **p < 0.01, ns= no significant compared to control. *Mann-Whitney test*.

### Interleukin-10 is the pivotal molecule connecting the effect of Pd in the gut with the skeletal muscle

We believe that the effect induced by postbiotic Pd supplement in skeletal muscle must be mediated by a factor secreted from the intestine and our proteomic study suggests that postbiotic Pd supplement induces a specific mitochondrial rewiring. Serum analysis shows that IL-10, a bona fide anti-inflammatory interleukin increases in postbiotic Pd supplemented mice (Fig 3D) [62]. Thus, to gain further insight, we determined whether postbiotic Pd induces the secretion of IL-10 from colon cell line HCT116. We treated the cells for 24h with Pd, collected the conditioned media and determined IL-10 levels by ELISA. As shown in Figure 7A, Pd increases the secretion of IL-10. Whether IL-10 affects mitochondrial function in skeletal muscle cells is unknown. Thus, we employed Seahorse technology to measure the oxygen consumption rate (OCR) in cultured skeletal muscle cells (HSkM) treated with recombinant IL-10 for 24 hours. Interestingly, both basal and maximal mitochondrial respiration were reduced following IL-10 treatment (Fig. 7B). Similarly, maximal respiration was also diminished in HSkM cells treated with conditioned media from HCT116 cells previously exposed to Pd (Fig. 7C) or when incubated with serum from Pd-treated mice (Fig. 7D).

Taken together, these findings suggest that postbiotic Pd supplementation induces IL-10 secretion in the intestine, which may subsequently reach skeletal muscle, where it modulates mitochondrial function.

## DISCUSSION

The global population of older adults (aged 80 years and above) is projected to increase by 200% by 2050 (World Population Ageing 2019, United Nations). Alongside this demographic shift, the world is experiencing an epidemiological transition characterized by a rise in chronic and degenerative diseases, including diabetes, hypertension, cardiovascular diseases, Parkinson’s, Alzheimer’s, and other cognitive disorders. This necessitates a deeper understanding of the aging process to enable effective interventions [63, 64]. Bacterial dysbiosis, the imbalance in the complex and dynamic population of microorganisms (bacteria, archaea, eukarya) colonizing the gastrointestinal (GI) tract, has been linked to several diseases, including those associated with aging [65–67]. It is well-documented that the gut microbial composition changes with age and correlates with markers of systemic and colonic inflammation. For instance, gut bacteria from aged mice increase the expression of proinflammatory genes when inoculated into the gut of young mice [68]. Conversely, the transfer of gut microbes from young killifish to middle-aged killifish significantly extends lifespan and delays behavioral aging, indicating that aging may be a modifiable process [69]. Furthermore, the physical decline and increased vulnerability observed in older adults, known as frailty, is associated with a reduction in the diversity of the microbiome [1]. This underscores the critical role of gut microbiota in aging and highlights the potential for microbiome-based interventions to mitigate age-related health issues.

The consumption of live bacteria (probiotics) to improve health has been part of human practice since antiquity and has gained significant popularity in the last two decades [70]. A growing number of probiotics with anti-aging potential have been identified. For instance, in elderly humans, *Lactobacillus casei* Shirota has been shown to increase serum levels of IL-10 and enhance the anti-tumor activity of natural killer (NK) cells [71]. Additionally, *Lactiplantibacillus plantarum* C29 has been reported to improve cognitive function [72]. In a mouse model of accelerated aging, *Lacticaseibacillus paracasei* PS23 was found to mitigate muscle mass and strength decline, and cognitive deterioration [73]. Although the anti-aging potential of *Parabacteroides distasonis* (Pd) was previously unknown, its presence, along with other *Parabacteroides* species, has been associated with a lower risk of infection in centenarians due to their production of secondary bile acids like lithocholic acid (LCA) [14]. The health benefits of Pd have been well-documented. For instance, Pd ameliorated rheumatoid arthritis by inhibiting the differentiation of Th17 cells and promoting M2 macrophage polarization [18]. Similarly, Pd protects against high-inflammatory non-alcoholic steatohepatitis (NASH) by generating pentadecanoic acid, which restores gut barrier integrity and reduces the expression of inflammatory cytokines [74]. Additionally, Pd alleviates liver fibrosis by increasing bile acid production [19] and has been shown to reduce inflammation and block colon tumor formation in high-fat diet-fed, azoxymethane-treated mice [17]. Our observations align with these findings, showing an increase in the anti-inflammatory cytokine IL-10 in serum and a reduction in colon shortening, typically caused by inflammation [60, 75], along with decreased levels of NFkβ in the colonic mucosa. In addition to its anti-inflammatory properties, Pd has been shown to modulate metabolism and mitigate obesity-related complications, including weight gain, hyperglycemia, and hepatic steatosis, in ob/ob and high-fat diet (HFD)-fed mice. These effects are mediated by the production of succinate and secondary bile acids in the gut [76]. Furthermore, nicotinic acid derivatives from Pd have been shown to activate G-protein-coupled receptor 109A (GPR109A) in the gut, leading to enhanced gut barrier integrity and improved insulin resistance [77]. Consistently, in our study, Pd supplementation resulted in reduced glycemia and decreased plasma lactate levels, which are known to increase with aging due to mitochondrial dysfunction and a shift toward glycolytic metabolism [78]. These findings suggest that Pd may exert beneficial metabolic effects by modulating mitochondrial function and energy metabolism.

Unlike previous studies, we used a postbiotic Pd preparation instead of live Pd. This suggests that a component of Pd is responsible for the anti-inflammatory effects, indicating that live interaction between Pd and the host is unnecessary. Postbiotics, which are preparations of inanimate microorganisms and/or their components [21], have been shown to provide a wide array of benefits, from reducing infantile colic to lowering cancer risk [79].

Interestingly, the components of our postbiotic Pd preparation were able to modify the host microbiome. Since the cecal samples were collected under aerobic conditions, it is possible that the DNA yield from less abundant anaerobic bacteria was reduced to levels below the detection threshold [80]. Despite this limitation, our results demonstrate that older mice fed with a postbiotic preparation of Pd developed a significantly distinct gut microbiota profile, characterized by a notable increase in health-beneficial bacterial genera, including *Alloprevotella, Bacteroides, Roseburia, Muribaculum, and Parasuterella* [81–85]. Concurrently, there was a decrease in *Ileiobacterium*, which has been associated with nonalcoholic fatty liver disease and high-fat diet-induced dysbiosis [60]. In rats, Pd also induced improvements in the microflora composition [86]. In the colonic cell line HCT116, we observed an upregulation of PGC1α, the master regulator of mitochondrial biogenesis, and an increase in mitochondrial networking and elongation. However, mitochondrial respiration was reduced. This outcome could be related to the presence of the bacterial metabolite butyrate, which is abundantly produced by *Bacteroides* and *Roseburia* [87, 88] and associated with *Alloprevotella* [89], all of which increased upon postbiotic Pd treatment. Butyrate exhibits similar effects on mitochondria [90, 91] by inhibiting histone deacetylases (HDAC), which increases the acetyl-CoA/CoA ratio, consequently inhibiting pyruvate dehydrogenase and reducing respiration. Butyrate can also mobilize intracellular calcium [92], which is known to regulate mitochondrial dynamics and function [93]. However, we cannot rule out the possibility that other metabolites produced by the newly induced microflora, or components of the Pd preparation itself, contributed to the observed modifications in mitochondrial function.

Postbiotic Pd supplementation improves and maintains grip strength in 18- and 26-month-old mice and enhances balance and coordination in 18-month-old mice. It is now well established that the gut influences skeletal muscle health through the gut-muscle axis [94], and both probiotics and postbiotics have been shown to improve muscle function. For example, the postbiotic *Lactiplantibacillus plantarum* TWK10 increases endurance and grip strength in healthy male adults [23]. Similarly, *Lacticaseibacillus paracasei* PS23, in both its probiotic and postbiotic forms, slows muscle strength loss and reduces fatigue in a model of exercise-induced damage [95]. Additionally, postbiotic preparations of *Ligilactobacillus gasseri* and *Lactococcus lactis* reduce skeletal muscle fatigue [96, 97].

Mitochondrial function is fundamental for skeletal muscle performance [98] and maintaining cellular homeostasis [99]. Aging is associated with mitochondrial dysfunction, characterized by increased reactive oxygen species (ROS) production, leading to oxidative damage to proteins, lipids, and mitochondrial DNA [100]. This initiates a vicious cycle in which mitochondrial impairment progressively worsens, further amplifying ROS generation. Skeletal muscle, which relies heavily on mitochondria to sustain energy homeostasis, is particularly vulnerable to mitochondrial dysfunction during aging, contributing to declines in muscle function and overall metabolic health [101].

Pd decreases respiration, which, in turn, reduces ROS generation while simultaneously triggering a pro-survival hormetic response as if the cells were under lower oxygen availability [102], as evidenced by an increase in CHOP. Consistent with this, our proteomic analysis reveals that mitochondrial-associated pathways are predominantly affected, with canonical pathways linked to mitochondrial dysfunction being downregulated, while those promoting cell survival are upregulated. VDAC2 downregulation is associated with reduced apoptosis, as this protein plays a crucial role in mitochondrial outer membrane permeabilization (MOMP) by interacting with the Bcl-2-associated X protein (BAX) and the Bcl-2 homologous antagonist/killer (BAK). This interaction facilitates cytochrome c release, a key event in the intrinsic apoptotic pathway [58, 59]. On the other hand, the increase in PGC1α and TFAM induced by Pd in skeletal muscle, as determined by Western blot and proteomics, alongside the increase in mitochondrial networking, suggests a rise in mitochondrial biogenesis. This increase in mitochondrial content may be linked to the reduced glycemia levels observed in 18- and 26-month-old mice and the tendency toward decreased lactate levels in 26-month-old mice. Additionally, this mitochondrial expansion may contribute to the improvement in skeletal muscle performance and the protection against high-fat diet-induced metabolic disturbances, as previously reported [103, 104].

We also sought to determine the messenger that communicates between the gut and the muscle. IL-10 plays a key role in maintaining muscle homeostasis [105] and increases upon postbiotic Pd treatment, as well as with other probiotics and postbiotics that positively impact skeletal muscle [71–73]. Interestingly, IL-10 can be synthesized and secreted by intestinal epithelial cells [106]. IL-10 has been shown to promote tissue growth and regeneration and improve the dystrophic phenotype in mdx mice by modulating macrophage polarization—specifically, by reducing M1 macrophage activation while concurrently enhancing M2 macrophage activity [107, 108]. Local injection of IL-10 in areas of volumetric muscle loss enhances tissue repair by modulating the immune response and promoting vascularization at the injury site [109]. Conversely, IL-10 homozygous knockout mice exhibit increased muscle weakness with age compared to wild-type mice [110]. In humans, IL-10 declines in circulation with age [111–113]. Notably, an IL-10 polymorphism associated with high serum levels of IL-10 is over-represented in centenarians, suggesting a link with longevity [114]. Recent reports demonstrate that the mitochondria of inflammatory macrophages can be metabolically reprogrammed by IL-10 [62, 115]. IL-10 was shown to mediate the induction of arginase-2 (Arg2) in mitochondria, skewing mitochondrial dynamics and bioenergetics in inflammatory macrophages by enhancing complex II activity of the electron transport chain [115]. Unlike macrophages, in skeletal muscle cells, we observed a reduction in mitochondrial respiration upon IL-10 stimulation, which was recapitulated with conditioned media or mouse serum enriched with IL-10. The mechanism responsible for this response remains unknown.

Mice and humans share approximately 90% similarity in gut microbiome composition at the phylum and genus levels. However, there are key differences in bacterial abundances, with humans generally exhibiting a higher *Firmicutes/Bacteroidetes* ratio compared to mice [116]. These differences introduce some uncertainty regarding the direct translation of our findings into human physiology. Nevertheless, Pd has been reported to be significantly reduced in individuals with obesity and metabolic syndrome [117, 118]—two conditions commonly associated with muscle dysfunction and decline [119, 120]. In patients with Crohn’s disease, decreased Pd abundance has also been observed alongside reduced muscle mass, although a direct relationship between these factors has not yet been established [121, 122]. Interestingly, Pd is well represented in the gut microbiome of centenarians, a population known for their exceptional resilience and extended healthspan [14]. Given these associations, it is plausible that a postbiotic Pd intervention may confer similar health benefits in humans as those observed in our mouse model, despite inherent microbiome differences. Future studies in human cohorts will be necessary to validate these findings and determine the translational potential of Pd-based interventions for metabolic and muscle-related disorders.

In summary, our data demonstrate that a postbiotic Pd-supplemented diet enhances overall health in aged mice. This effect is mediated, at least in part, by an increase in IL-10 levels and mitochondrial adaptations that may trigger a mitohormetic response, opening new avenues for the treatment of age-related diseases.

## Supplementary Materials

The Supplementary data can be found online at: www.aginganddisease.org/EN/10.14336/AD.2025.0188.
