# Supplementary material for: Postbiotic Parabacteroides Distasonis Supplementation Enhances Intestinal and Skeletal Muscle Function in Aged Mice
Source: Aging Dis. 2025 Apr 22;17(3):1534–56. doi: 10.14336/AD.2025.0188 (PMC13061540; doi:10.14336/AD.2025.0188)
Supplement: Supplementary file 1 — The Supplementary data can be found online at: www.aginganddisease.org/EN/10.14336/AD.2025.0188. [file ad-17-3-1534-s.pdf]

SUPPLEMENTARY DATA

Postbiotic *Parabacteroides Distasonis* Supplementation Enhances Intestinal and Skeletal Muscle Function in Aged Mice

Pablo Morgado-Cáceres, Hernán Huerta, Cristian Bergman, Reinaldo Figueroa, Paula Farias, Gabriel Quiroz, Ute Woehlbier, Karen Mella, Osmán Díaz-Rivera, Sergio Linsambarth, Paulina Calderón-Romero, Felipe A. Court, Denisse Sepulveda, Daniela Sauma, Patricia Luz-Crawford, Anibal A. Vargas, Catalina Gonzalez-Seguel, J. César Cárdenas, Alenka Lovy

© 2025. Morgado-Cáceres P. et al. Published online at http://www.aginganddisease.org/EN/10.14336/AD.2025.0188

SUPPLEMENTARY DATA

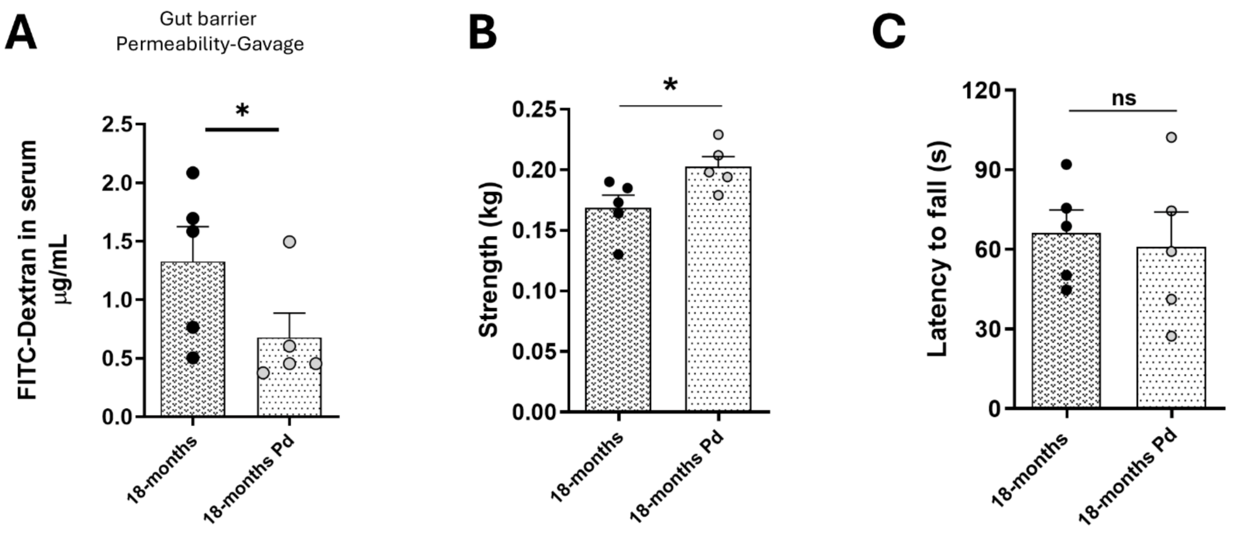

Supplementary Figure 1. Postbiotic Pd supplementation via gavage enhances gut barrier integrity, strength, and motor coordination. 18-month-old mice received a weekly gavage of 10 mg lyophilized postbiotic *Parabacteroides distasonis* (Pd) in 200 µL of PBS for three months. Control mice received a weekly gavage of PBS. **S1A.** Following the Pd treatment period, mice were gavage-fed FITC-dextran (0.6 mg/g body weight), and fluorescence intensity in the serum was measured 4 hours later as an indicator of gut barrier permeability. N = 5 for the Pd treated group and 6 for the control. Data are presented as MEAN ± SEM, $p < 0.05$*, Mann-Whitney test.* **S1B**. Fore-/hindlimb (4 paws) grip strength was determined following Pd treatment, N = 5 for the Pd treated group and 6 for control group. Data are expressed as MEAN ± SEM. *$p \leq 0.05$, *Mann-Whitney test*. **S1C**. Latency to fall on accelerating rotarod was performed after the Pd treatment. N = 5 for the Pd treated group and 6 for the control. Data are presented as MEAN ± SEM, ns=not significant, *Mann-Whitney test*.

© 2025. Morgado-Cáceres P. et al. Published online at http://www.aginganddisease.org/EN/10.14336/AD.2025.0188
